# Mg-HA-C/C Composites Promote Osteogenic Differentiation and Repair Bone Defects Through Inhibiting miR-16

**DOI:** 10.3389/fbioe.2022.838842

**Published:** 2022-02-04

**Authors:** Hong Qi, Yang Liu, Lu Wu, Chun Liu, Su Ni, Qizhan Liu, Xinye Ni, Qiang Sun

**Affiliations:** ^1^ Center for Global Health, The Key Laboratory of Modern Toxicology, Ministry of Education, School of Public Health, Nanjing Medical University, Nanjing, China; ^2^ Department of Orthopedics, Nanjing First Hospital, Nanjing Medical University, Nanjing, China; ^3^ Second People’s Hospital of Changzhou, Nanjing Medical University, Changzhou, China

**Keywords:** miR-16, hydroxyapatite, C/C composite, magnesium, bone regeneration

## Abstract

The hydroxyapatite (HA) coating on carbon/carbon (C/C) is reasonable and feasible to obtain bone graft materials with appropriate mechanical and biological properties. However, improvement of the physical and chemical properties of HA-C/C composites to promote bone regeneration and healing remains a challenge. In our present study, the HA coatings on C/C with magnesium (Mg) (Mg-HA-C/C) composites were synthesized that Ca (NO_3_)_2_, Mg (NO_3_)_2_, and NH_4_H_2_PO_4_ were mixed and coatings were made by electromagnetic induction deposition’s heating. As determined with *in vitro* experiments, Mg-HA-C/C composites containing 10 and 20% Mg decreased miR-16 levels, increased cell viability, elevated the levels of osteogenesis-related genes, and promoted osteogenic differentiation of bone marrow mesenchymal stem cells (BMSCs) seeded on their surfaces. In a rat model of skull defects, compared to the control group, at 4 and 12 weeks after the operation, the bone volume fraction (BV/TV) of Mg-HA-C/C composite group was increased by 8.439 ± 2.681% and 23.837 ± 7.845%, as well as the trabecular thickness (Tb.Th) was 56.247 ± 24.238 μm and 114.911 ± 34.015 μm more. These composites also increased the levels of ALP and RUNX2 in skull. The Mg-HA-C/C composite-enhanced bone regeneration and healing were blocked by *in situ* injection of an miR-16 mimic lentivirus vector. Thus, Mg-HA-C/C composites promote osteogenic differentiation and repair bone defects through inhibiting miR-16.

## 1 Introduction

MicroRNAs (miRNAs), which are non-coding RNAs, are not translated into proteins. Among the non-coding RNAs, miRNAs, with approximately 22 nucleotides, are relatively short (Ambros, 2004). The seed sequences of miRNAs recognize the complementary sequence of the 3′untranslated region (UTR) of their target mRNA to degrade the target mRNA or to inhibit its protein translation (Ha and Kim, 2014; Peng and Croce, 2016). miRNAs are involved in various cellular processes, such as self-renewal, proliferation, function, and differentiation (Garcia and Delany, 2021). By regulating the TGF-β and BMP signaling pathways of BMSCs, miRNAs guide their development to the osteoblast lineage. These pathways are involved in the process of bone formation and bone healing (Bai et al., 2019). An understanding of the mechanisms for miRNA-mediated induction of pluripotent cells in osteoblasts is necessary for effective use of miRNAs as therapeutic candidates for bone diseases such as osteoporosis (Garcia and Delany, 2021). The low expression of miR-16-5p promotes the osteogenic differentiation of hMSCs by up-regulation of VEGFA (Yu et al., 2020). In our previous study, we confirmed that Mg ions promoted the osteogenic differentiation of BMSCs through the regulation of miRNA-16 (Qi et al., 2020). Therefore, whether Mg containing biomaterials promote bonee formation and healing by mediating miR-16 deserves to be studied.

Due to the influence of diseases, aging, accidents, and other factors, cases of bone defects are increasing (Wang C. et al., 2019; Oza et al., 2020). In recent years, hydroxyapatite (HA), a representative bone substitute material, has been used in clinical applications. HA, the main inorganic component of human and animal bones and teeth, has appropriate bioactivity and biocompatibility (Dinda et al., 2020; Shen et al., 2020). Compared with traditional metals (e.g., stainless steel, titanium alloy) and ceramic (e.g., alumina, silicon nitride) bone substitutes, HA has strong corrosion resistance and bone induction capacity, and it eliminates the safety risks of traditional materials by its degradation *in vivo* (Beh et al., 2020; Feng et al., 2020). Carbon/carbon (C/C) composites and surface-modified bone repair materials have fracture toughness. They also have and an elastic modulus equivalent to that of human bone, and they promote cell biocompatibility (Chen et al., 2018; Hashemi and Rezania, 2019; Wu Y. et al., 2020; Ge, Veksha and Lisak, 2020; Yevtushenko et al., 2020; X. Zhang et al., 2020). Accordingly, they have recently become a feature of biomedical bone repair and replacement biomaterials. However, pure HA dissolves in physiological environments, and its instability often causes implant failures (Xin-Bo et al., 2016).

In natural bone, the mass percentage of magnesium (Mg) ranges from 0.26 to 0.55%. The concentration of Mg ions affects the calcification process of human bone (de Baaij et al., 2015; J. Zhang et al., 2019). From the beginning to the completion of calcification, the concentration of Mg ions changes from high to low (DiNicolantonio, O'Keefe and Wilson, 2018). Mg prevents osteoporosis, regulates immune function, and promotes the formation of HA crystals; mineral calcification deposition; and bone cell adhesion, proliferation, and differentiation (Castiglioni et al., 2013; Jang et al., 2014; Abdallah et al., 2016). The concentration of Mg in serum is negatively correlated with osteoporosis (He et al., 2021). The reduction of Mg ions can affect the bone metabolism process by inhibiting the activity of osteoblasts and osteoclasts, thus resulting in osteopenia (Rude et al., 2009; Hohenbild et al., 2021). In recent years, many studies have found that adding Mg to various biomaterials can promote the healing and regeneration of bone tissue (Li et al., 2021; Tamay et al., 2021). Pure Mg degrades quickly under physiological conditions and releases hydrogen into the surrounding fluid environment, which limits its research and clinical applications in orthopedics (Wu Z. et al., 2020). In our previous study, we found that MgCl_2_ promotes osteogenic differentiation of bone marrow mesenchymal stem cells (BMSCs) by activating the ERK/MAPK pathway (Qi, et al., 2020). High-purity Mg bone screws and interface screws have a good osteogenic calcification effect in fracture stress and bone tunnels; this is achieved by the activation, by Mg ions, of bone morphogenetic protein-2 (BMP-2) and vascular endothelial growth factor (VEGF) (Han et al., 2015; Cheng et al., 2016). Therefore, HA could be doped with Mg to improve its bio-performance.

In the present investigation, the research on HA in bone substitute materials mainly focused on the positive effect of coating C/Cs with Mg composites for bone regeneration and on elucidating the underlying molecular mechanism. We designed Mg-doped HA coating on the surface of C/C composites and evaluated its biocompatibility and promoting effect on bone regeneration through *in vitro* and *in vivo* tests. This information will be helpful for the clinical application of this material.

## 2 Materials and Methods

### 2.1 Synthesis of Mg-Doped Hydroxyapatite Coating on Carbon/carbon Composites

The Mg-doped HA coating on C/C composites were synthesized following the protocol previous described (Xinbo et al., 2016). Briefly, the 2.5D PAN-based C/C composites (Yixing Tianniao High Tech Co., Ltd, China) with 182.3 MPa bending strength, 90.4 GPa bending modulus, and 35.0 GPa elastic modulus were turned into a cylinder (1.2 cm in diameter and 0.6 cm in height). Then, the cylinder was deposited by induction heating by ZAG-15 kW Hi-Fi machine (Wujin Zheng’ao High-frequency Machinery Factory, China). Ca (NO_3_)_2_, Mg (NO_3_)_2_, and NH_4_H_2_PO_4_ were mixed in distilled water. After 2 h of electromagnetic induction and deposition, coating and subsequent studies were conducted. HA-C/C composites containing no Mg were considered as the control.

### 2.2 Material Characterization

The morphology and composition of the samples were characterized by scanning electron microscopy (SEM) and energy dispersive spectroscopy (EDS) (S-3400N, Hitachi High Technologies Co., Tokyo, Japan). In the Mg ion release experiment, a single piece of material was put into 50 ml DMEM medium and incubated in a 37°C incubator. After 1, 3, and 6 days, the concentrations of Mg ion in the culture medium were measured by vista-ax plasma emission spectrometer.

### 2.3 *In vitro* Cellular Studies

#### 2.3.1 Culture of Bone Marrow Mesenchymal Stem Cells

BMSCs were separated from femurs and tibias of Sprague−Dawley (SD) rats (3 weeks old) by flushing out the bone marrow with complete medium (Dulbecco’s modified Eagle’s medium (DMEM; Gibco, MA, United States) supplemented with 10% fetal bovine serum (FBS; Gibco, MA, United States) and 1% (v/v) penicillin/streptomycin (Gibco, MA, United States). Then the cells were cultured at 37°C in a 5% CO_2_ incubator. BMSCs were passaged at a confluence of 80–90% and passages 2–6 were used for the experiments.

BMSCs were seeded on the surfaces of HA-C/C composites containing various concentrations (0%, 10%, or 20%) of Mg, which were made into circular thin sheets (10 mm × 2 mm; 1 × 10^5^ cells) and cultured for 72 h. The cells were transfected with green fluorescent protein (GFP) and then imaged with a fluorescence microscope (CLSM; LSM 510, Zeiss).

#### 2.3.2 Cell Viability Assay

The viability of BMSCs grown on the Mg-HA-C/C composites containing different concentrations (0%, 10%, or 20%) of Mg was determined using CCK-8 kits (Dojindo, Japan). BMSCs (1 × 10^5^) were seeded on the surfaces of the materials and cultured for 72 h. Then the medium was replaced with 500 μl of fresh medium containing 10% CCK-8 solution in each well. After 2 h of incubation, portions (100 lL) from each well were placed in a 96-well plate for measurement. The absorbance of the samples was measured at a wavelength of 450 nm using a microplate reader (Bio-Rad 680, CA, United States).

#### 2.3.3 RNA Preparation and Quantitative Real-Time Polymerase Chain Reaction

BMSCs were seeded on the surfaces of HA-C/C composites containing various concentrations (0%, 10%, or 20%) of Mg with a density of 2 × 10^5^ cells/sheet and cultured for 7 days. Then total RNA was isolated by homogenizing the scaffolds with cells in 1 ml of Trizol reagent (Invitrogen Life Technologies Co.,CA, United States). The bone of the rat skull transplantation area was repeatedly ground to powder shape, and Trizol was added. After full homogenization, centrifugation at 4°C. Complementary DNA (cDNA) was obtained using a PrimeScript first Strand cDNA Synthesis kit (Takara, Japan) following the manufacturer’s instructions. To detect miRNAs, 1 μg of total RNA and HiScript II Q Select RT Supermix (Vazyme biotech, Nanjing, China) were used in reverse transcription. Quantifications of cDNAs of alkaline phosphatase (ALP), runt-related transcription factor 2 (RUNX2), osterix (Sp7), osteocalcin (OCN), osteopontin (OPN), and miR-16 were performed with an ABI7500 Thermal Cycler (Applied Biosystem, Australia) using real-time PCR kits (SYBR Premix EX Taq, Takara). All assays were performed in triplicate. The primers used were presented in Table 1.

**TABLE 1:** Primer sequences used.

*GAPDH*	*F: 5′-GCA​TCC​TGG​GCT​ACA​CTG-3′*
	*R: 5′-TGG​TCG​TTG​AGG​GCA​AT-3′*
*ALP*	*F: 5′-GGT​CAC​CAG​GGC​TGC​TTT​TA-3′*
	*R: 5′-GGA​TCT​CGC​TCC​TGG​AAG​ATG-3′*
*OCN*	*F: 5′-CCA​CGT​CTT​CAC​ATT​TGG​TG-3′*
	*R: 5′-AGA​CTG​CGC​CTG​GTA​GTT​GT-3′*
*RUNX2*	*F: 5′-CAT​GAG​GAC​CCT​CTC​TCT​GC-3′*
	*R: 5′-TGG​ACA​TGA​AGG​CTT​TGT​CA-3′*
*Sp7*	*F: 5′-TGT​CAT​GGC​GGG​TAA​CGA​T-3′*
	*R: 5′-AAG​ACG​GTT​ATG​GTC​AAG​GTG​AA-3′*
*OPN*	*F: 5′-GAG​GCA​ACT​GGC​TAG​GTG​G-3′*
	*R: 5′-CTG​GAT​TAA​GGG​GAG​CAA​AGT​C-3′*

#### 2.3.4 Alkaline Phosphatase Staining and Alizarin Red S Staining

BMSCs in growth culture medium were seeded in 24-well plates at a density of 7 × 10^4^ cells/well. When their confluence reached 60%, BMSCs were treated with osteo-inductive medium containing an extract of Mg-HA-C/C composites for 14 days. After that, ALP staining and alizarin red S staining were performed as described (Qi, et al., 2020).

### 2.4 *In vivo* Bone Regeneration of the Magnesium-Hydroxyapatite-Carbon/carbon Composites

#### 2.4.1 Animal Surgical Procedures

40 female SD rats (8 weeks old, 200–250 g) used in the animal experiments were randomly divided into four groups: group 1, rats were implanted with the HA-C/C composite on both sides; group 2, rats were implanted with the HA-C/C composite on the right side, the Mg-HA-C/C composite was implanted on the left side; group 3, rats were planted with the HA-C/C composite on the right side, the Mg-HA-C/C composite was implanted on the left side and GFP-labeled miR-16 mimic lentivirus vector was injected into the implanted site; group 4, rats were implanted with the HA-C/C composite on the right side, the Mg-HA-C/C composite on the left side, and GFP-labeled miR-16 con was injected into the implanted site. Following anesthesia, a sagittal incision (1.0–1.5 cm) was prepared on the scalp, and the calvarium was exposed by blunt dissection. Two defects with a diameter of 5 mm were created with an electric trephine (Nouvag AG, Goldach, Switzerland) under constant irrigation with normal saline. The defects were implanted with a disc of HA (diameter 5 mm). For each group, pure HA was implanted on the right side of the defect, and HA with various treatments was transplanted on the left side. Finally, the incisions were closed with absorbable sutures. Each rat received an intramuscular injection of penicillin post operation. At the 4th and 12th week, five animals in each group were killed, and their skulls were taken for examination.

#### 2.4.2 Microcomputed Tomography Assessment

At 4 or 12 weeks post-operation, a micro-CT device (Skyscan 1176, Kontich, Belgium) was used to scan undecalcified calvaria at a resolution of 18 μm to evaluate the formation of new bone in the defects. Three dimensional (3-D) images were reconstructed with the CTVox program (Skyscan). Values for newly formed bone volume to total volume (BV/TV) and trabecular thickness (Tb.Th) were obtained by the CTAn program (Skyscan). The bone formation capacity of different groups was compared by statistical analysis of the difference of the data for the two sides of the rat skull.

#### 2.4.3 Bone Tissue Imaging With Fluorescence Labeled miRNA

After implantation, a fluorescent-labeled miR-16 mimic or a miRNA control (miR-con) lentivirus vector was injected into sides with the Mg-HA-C/C composite *in situ*. At 4 weeks post-operation, the images were generated using an IVIS Spectrum (Caliper).

#### 2.4.4 Histological Evaluation

For histological evaluation and osteogenic investigation, the skulls were immersed in formalin buffer solution, and the remaining skulls of the different groups were decalcified with ethylenediaminetetraacetic acid disodium solution (12%, v/v) for 28 days. The fixed skulls were dehydrated with gradient ethanol solutions, cleared with xylene, and embedded in polymethyl methacrylate (PMMA). Thereafter, a histological section perpendicular to the implant was derived by microtome (Leica SP1600, Germany) (approximately 5 μm for soft-cut samples and approximately 50 μm for hard-cut samples). Hematoxylin-eosin (HE) staining, Masson staining, and immunohistochemistry of ALP and RUNX2 were performed.

### 2.5 Statistical Analysis

All data were expressed as means ± SEM. One-way analysis of variance (ANOVA) and paired or unpaired two-tailed Student’s t tests were conducted using Prism (GraphPad7). *p* values of statistical significance were represented as *, #, or and *p* < .05.

## 3 Results

### 3.1 Analysis and Characterization of Hydroxyapatite-Carbon/carbon and Magnesium-Hydroxyapatite-Carbon/carbon Composites

**FIGURE 1:**
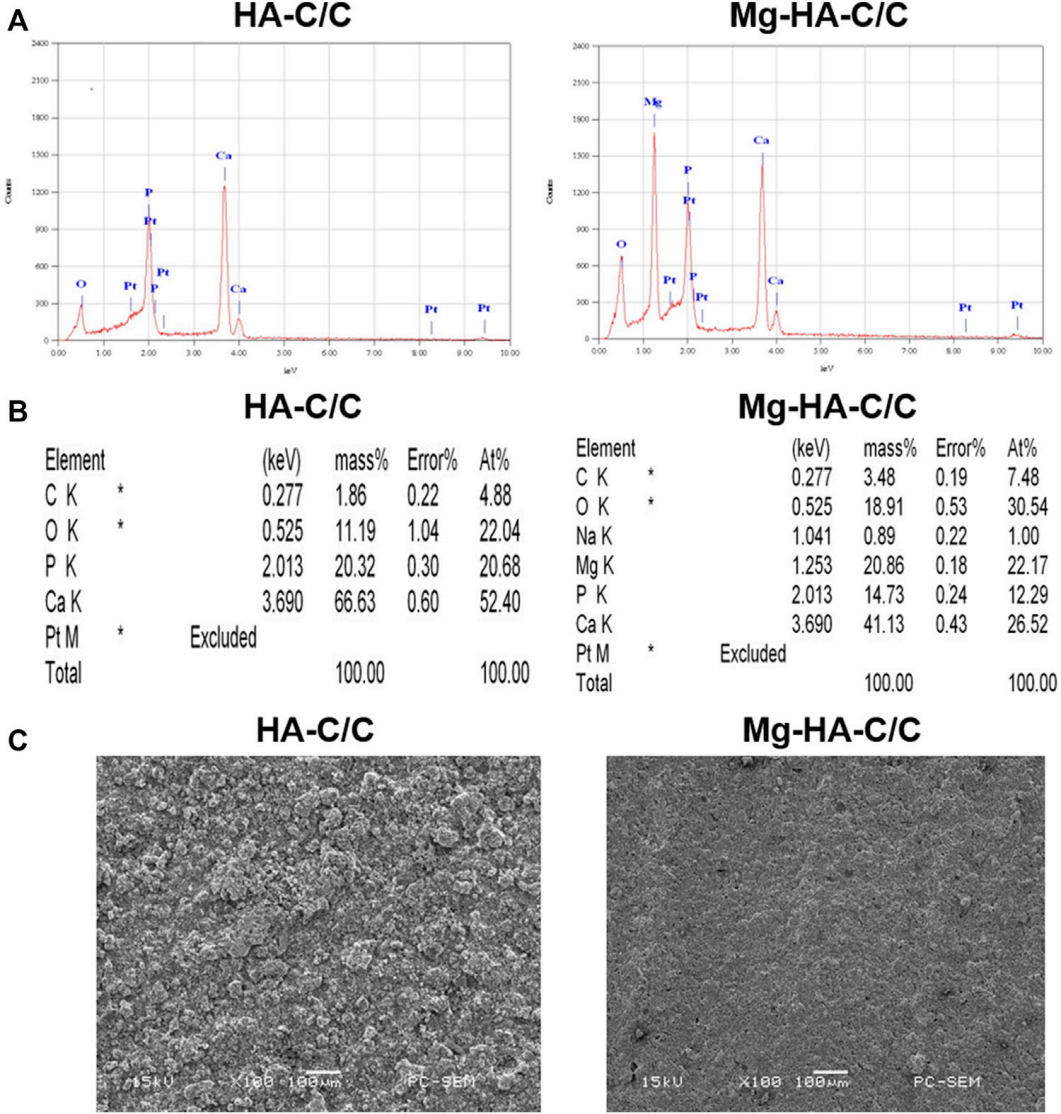
The characterization of HA-C/C and Mg-HA-C/C composites. EDS spectra **(A)**, element intensity **(B)**, and SEM images **(C)** of HA-C/C and Mg-HA-C/C composites.

We prepared Mg-HA coatings on the surface of C/C composites by chemical vapor deposition. The physicochemical characteristics of the HA-C/C and Mg-HA-C/C composites are shown in Figure 1. The EDS spectra of HA-C/C and Mg-HA-C/C composites were derived; the results showed the presence of Mg, Ca, Pt, O, C, and P in the HA phases (Figures 1A,B). The Mg-HA-C/C composites contained Mg; pure HA coatings had none. For the pure HA coatings, there were large particles on the surface of C/C composites as determined by SEM images, however; for the Mg-HA coatings, there were only small particles, which constructed a much denser surface than that of the pure HA coating (Figure 1C). To detect the release of Mg ions from our Mg-HA-C/C composites, we placed Mg-HA-C/C composites with 0%, 10%, or 20% Mg contents in DMEM culture solution, and the concentrations of Mg ions in the solution were measured by vista-ax plasma emission spectrometer at 0, 1, 3, or 6 days. Data showed that the concentrations of Mg ions in the solution Mg-HA-C/C with 10% Mg and 20% Mg were higher than those in the solution Mg-HA-C/C with 0% Mg at 1, 3, or 6 days (Supplementary Table S1). Therefore, because of their relatively more compact surface and the release of Mg ions, Mg-HA-C/C composites may promote bone reconstruction and healing.

### 3.2 Magnesium-Hydroxyapatite-Carbon/carbon Composites Increase Cell Viability, Decrease miR-16 Levels, and Promote the Osteogenic Differentiation of BMSCs *in vitro*

**FIGURE 2:**
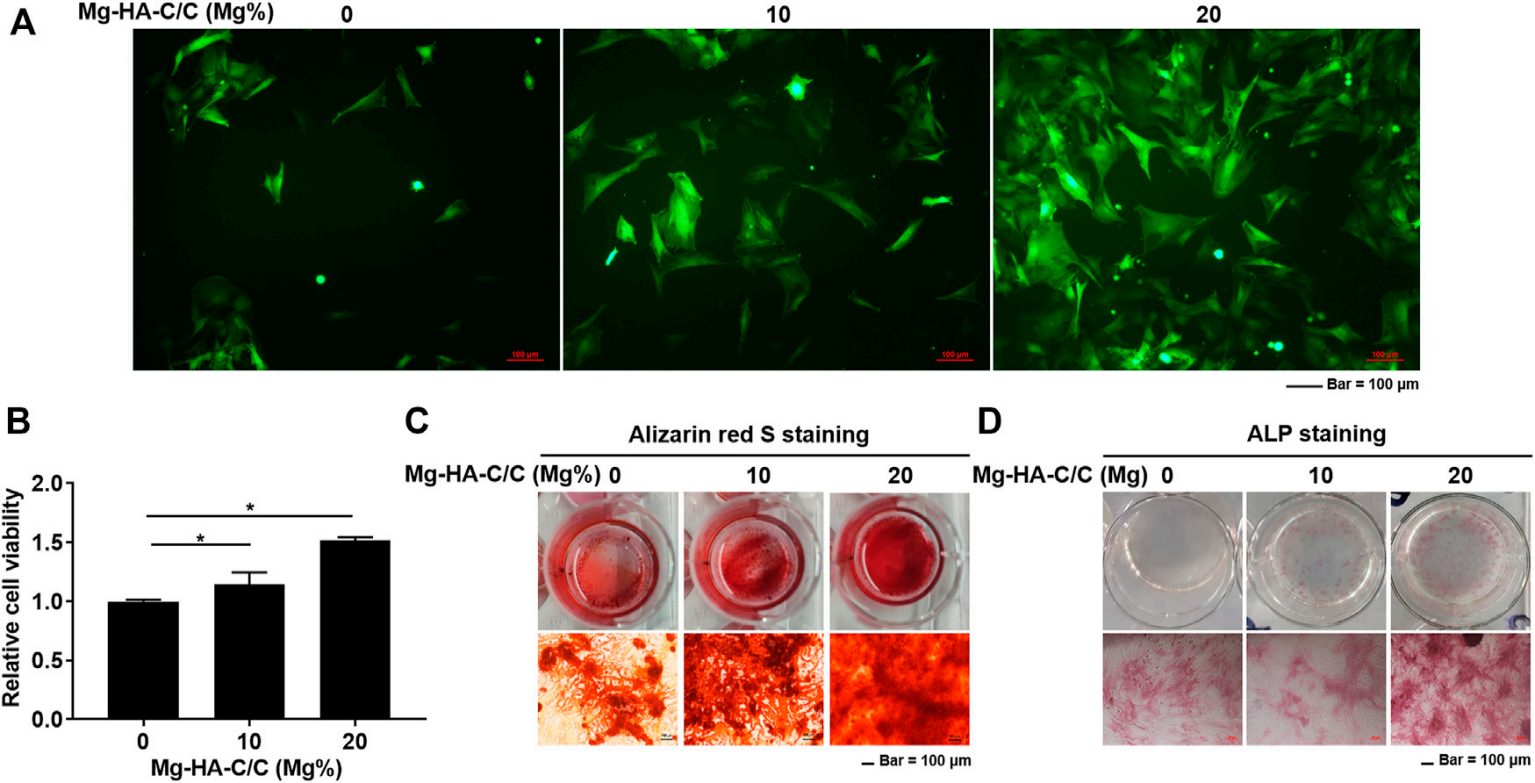
Mg-HA-C/C composites increase viability and promote the osteogenic differentiation of BMSCs *in vitro*. BMSCs were labeled with GFP and cultured on the surfaces of Mg-HA-C/C composites with 0%, 10%, or 20% Mg contents. **(A)** Morphological images of BMSCs were observed by fluorescence microscopy (Bar = 100 μm). **(B)** The viability of BMSCs was assessed with CCK8 kits (mean ± SD, *n* = 6), *p < .05. BMSCs were subjected to osteogenic differentiation in medium containing soaking liquid of Mg-HA-C/C composites. The alizarin red S staining **(C)** and ALP staining **(D)** were performed (Bar = 100 μm).

BMSCs were cultured to investigate the effect of Mg-HA-C/C composites on their capacity for osteogenic differentiation. BMSCs were seeded on the surfaces of HA with coatings with various Mg contents (0%, 10%, or 20%). A lentiviral vector containing GFP was transfected into BMSCs, and growth of the cells was observed under a fluorescence microscope. With high Mg contents in HA coatings, more BMSCs were present (Figure 2A). The viability of BMSCs on the surface of Mg-HA coatings was greater than that of those on surfaces without Mg (Figure 2B). To confirm the effect of Mg-HA coatings on osteogenic differentiation, extracts of HA-C/C composites with different Mg contents were used to treat BMSCs. A greater mineralization deposition and higher ALP content was evident for the cells treated with extracts of Mg-HA coatings (Figures 2C,D).

**FIGURE 3:**
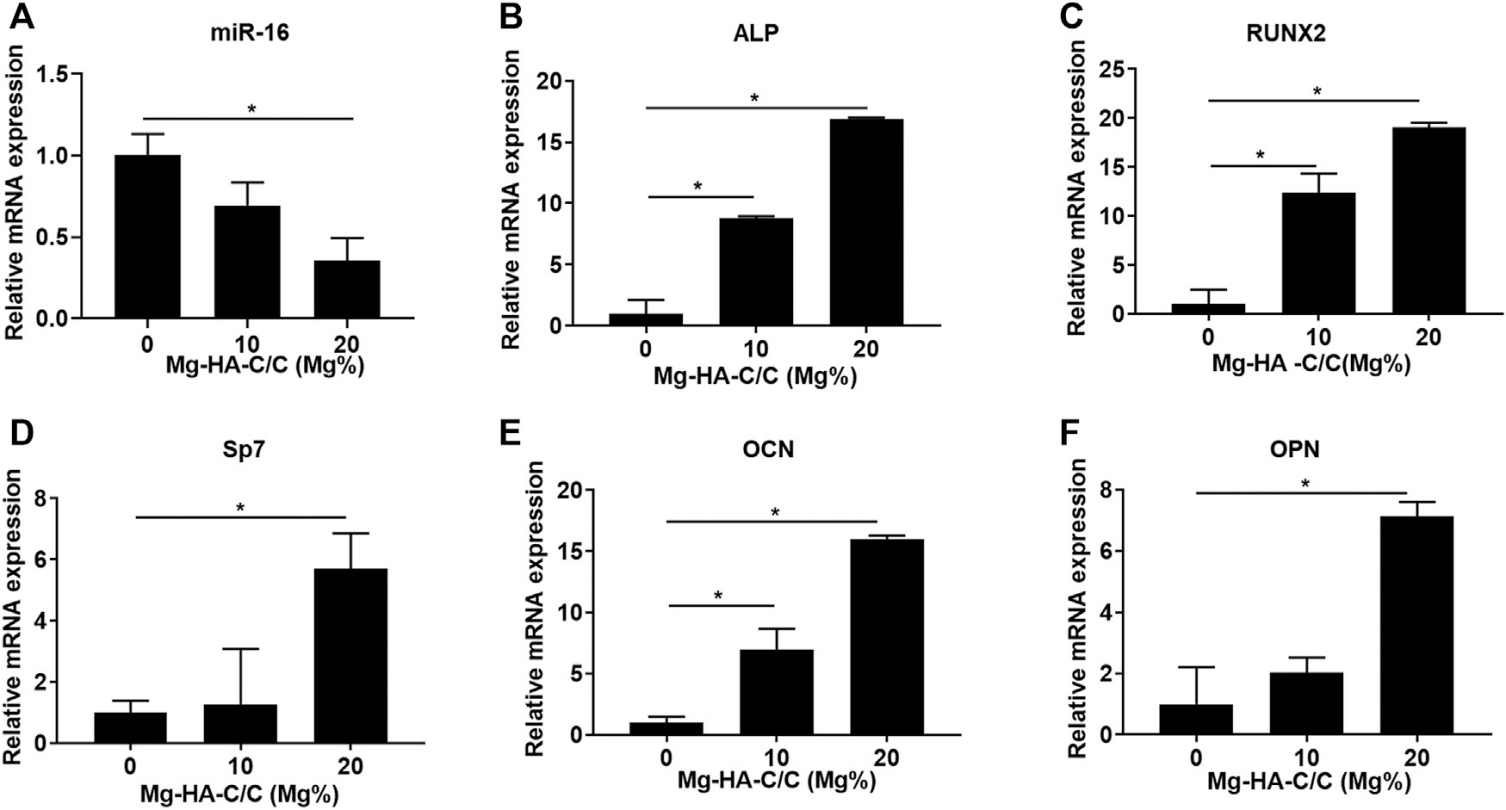
Mg-HA-C/C composites increase the expression of genes related to osteogenic differentiation *in vitro*. BMSCs were seeded on the surfaces of Mg-HA-C/C composites with 0%, 10%, or 20% Mg contents and then were induced to osteogenic differentiation. The levels of miR-16 **(A)** and genes related to osteogenic differentiation ***(*** *ALP*, *RUNX2*, *Sp7*, *OCN*, and *OPN)* **(B–F)** were measured by qRT-PCR (mean ± SD, *n* = 6). ^*^ *p* < .05.

RNA from BMSCs growing on the surfaces of HA with various Mg contents was extracted to assess the expression of miR-16 and osteogenic differentiation-related genes. The expression of miR-16 was lower for the groups treated with Mg-HA-C/C composites (Figure 3A). Moreover, the levels of ALP, RUNX2, Sp7, OCN, and OPN were higher in the BMSCs growing on surfaces of HA containing Mg (Figures 3B–F). These results suggest that Mg-HA-C/C composites increase cell viability and promote the osteogenic differentiation of BMSCs *in vitro*.

### 3.3 Magnesium-Hydroxyapatite-Carbon/carbon Composites Promote Bone Formation and Healing *in vivo*

**FIGURE 4:**
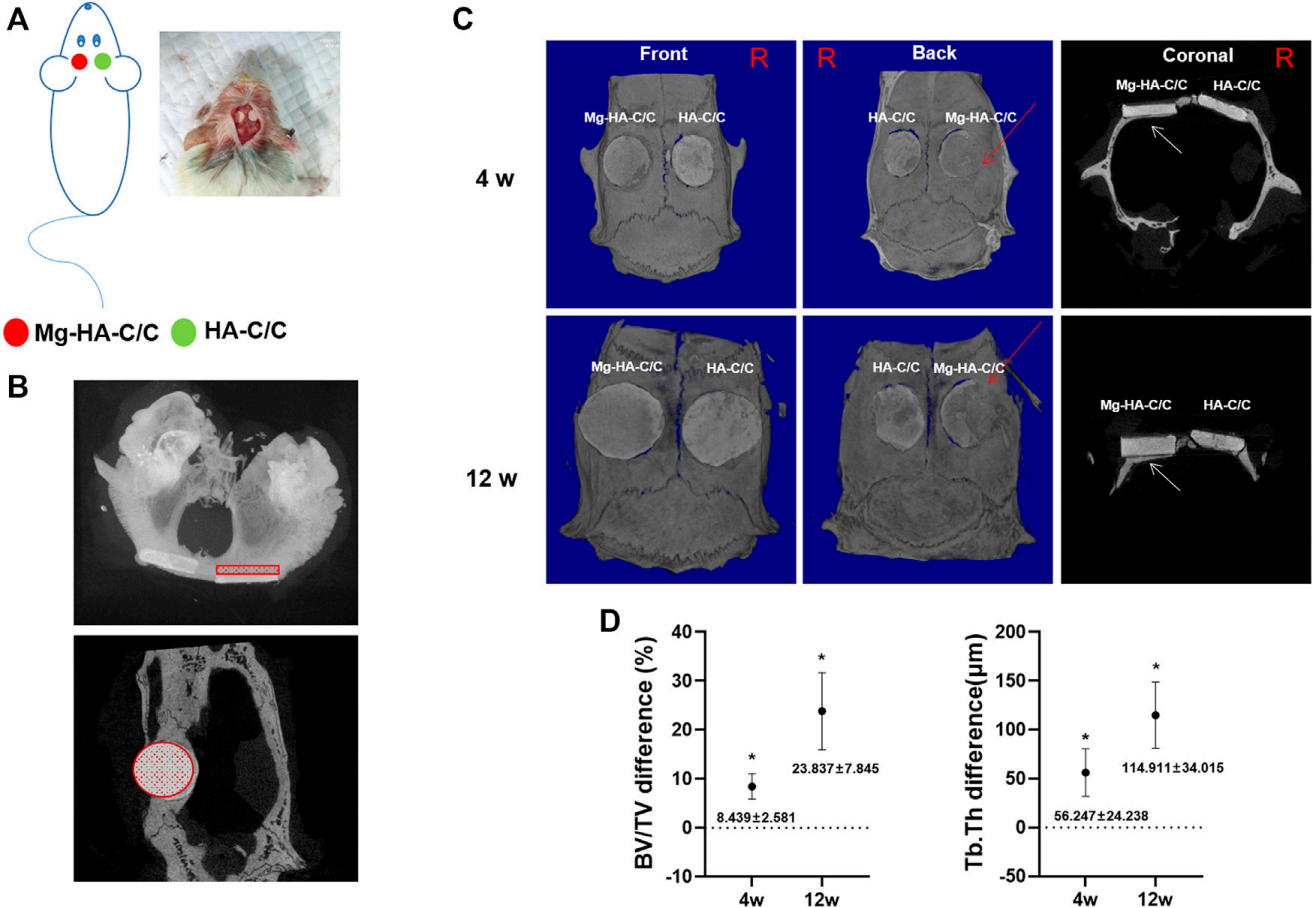
Mg-HA-C/C composites promote bone formation *in vivo* as determined by micro-CT analysis. The HA-C/C or Mg-HA-C/C composites were implanted on the skulls, and the rats were maintained for 4 or 12 weeks. **(A)** Schematic graph illustrating the animal experiment. **(B)** Cross and coronal images showed the area of the skull selected for micro-CT analysis. **(C)** Representative 3D-reconstructed micro-CT images and coronal images were acquired. The red arrows on 3D reconstruction and the white arrows on coronal images indicate new bone formation. **(D)** The BV/TV and Tb.Th differences were calculated by CTAn analysis software (mean ± SD, *n* = 5). ^*^ *p* < .05.

To investigate the effect of Mg-HA-C/C composites on repair of bone defects, we implanted HA-C/C and Mg-HA-C/C composites into rats with holes in their skulls. On the skulls, HA without Mg coatings were implanted in the left hole, and HA with Mg coatings were implanted in the right hole (Figure 4A). Bone regeneration occurred on the inner side of the skulls; for the outer side, bone regeneration was not obvious. Therefore, to quantify bone formation, we selected a round section of 5 mm diameter inside the skull of each rat, sliced it at 18 microns, and evaluated 25 consecutive pieces (Figure 4B). Micro-CT showed that bone regeneration of the side with Mg-HA-C/C composites was, at 4 and 12 weeks after the operation, higher than that for composites without Mg, including the BV/TV of Mg-HA-C/C composite group was increased by 8.439 ± 2.681% and 23.837 ± 7.845%, as well as the Tb.Th was 56.247 ± 24.238 μm and 114.911 ± 34.015 μm more (Figures 4C,D; Supplementary Video S1).

**FIGURE 5:**
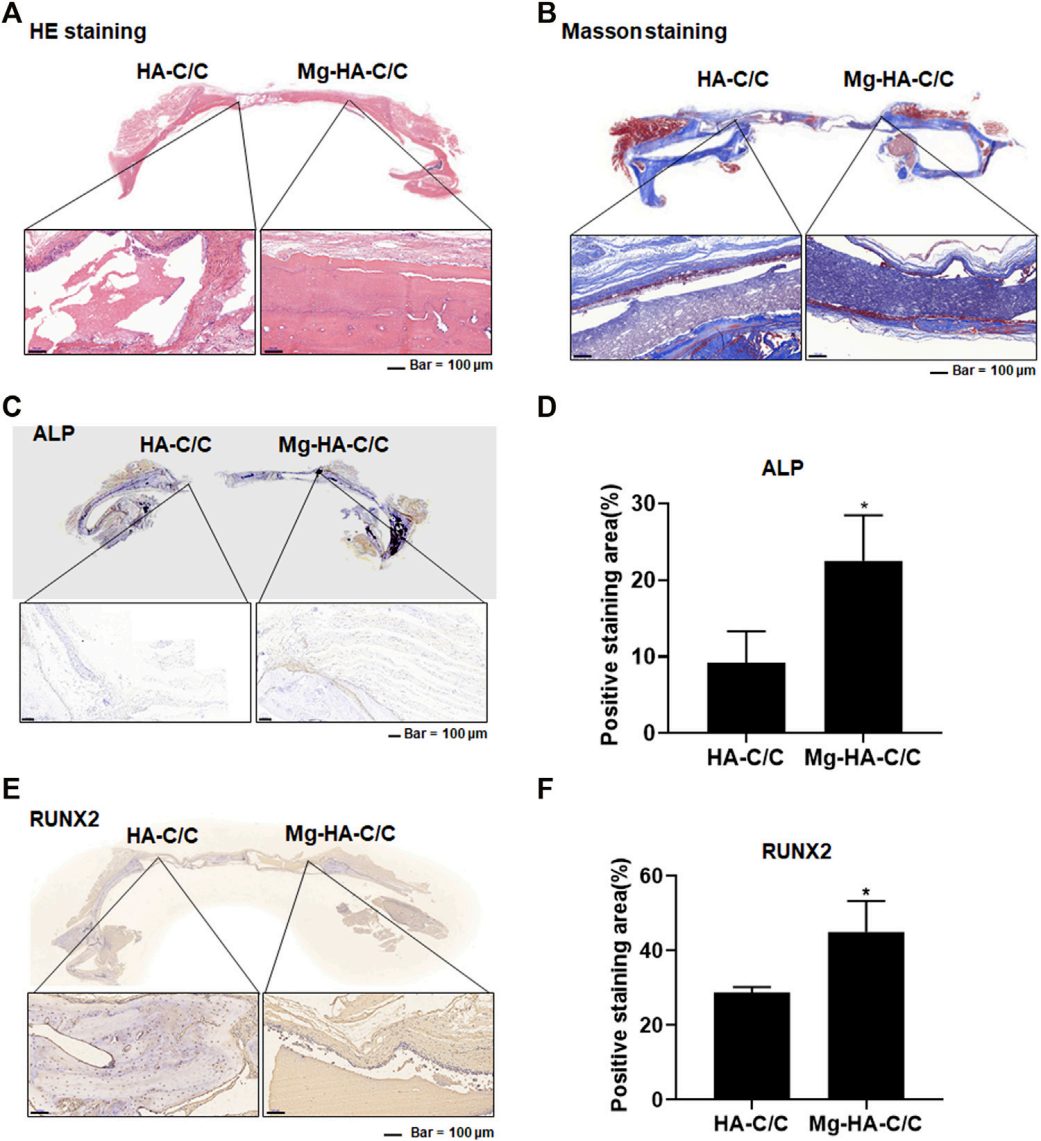
Mg-HA-C/C composites promote bone formation and increases of ALP and RUNX2 levels in skull tissues. The HA-C/C or Mg-HA-C/C composites were implanted on the skulls, and the rats were maintained for 4 weeks. HE staining **(A)** and Masson staining **(B)** of the skulls were performed. **(C)** IHC staining of ALP was accomplished, and **(D)** the positive staining areas were quantified in skull tissues of rats. **(E)** IHC staining of RUNX2 was performed, and **(F)** the positive staining areas in skull tissues of rats. mean ± SD, *n* = 5. ^*^ *p* < .05.

Results of the histological and immunohistochemical analyses of bone regeneration are shown in Figure 5. HE staining revealed bone regeneration in the defect of the side with the Mg-HA-C/C composites (Figure 5A). Masson staining also showed more collagen regeneration in the bone defect side with the Mg-HA-C/C composite (Figure 5B). Further, immunohistochemical staining showed higher levels of RUNX2 and ALP in the sides with Mg-HA-CC composites compared with those with pure HA coatings (Figures 5C–F). These results indicate that Mg-HA-C/C composites promote bone formation and healing in rats.

### 3.4 Overexpression of miR-16 Blocks the Enhancement of Bone Formation and Reconstruction Induced by Magnesium-Hydroxyapatite-Carbon/carbon Composites

**FIGURE 6:**
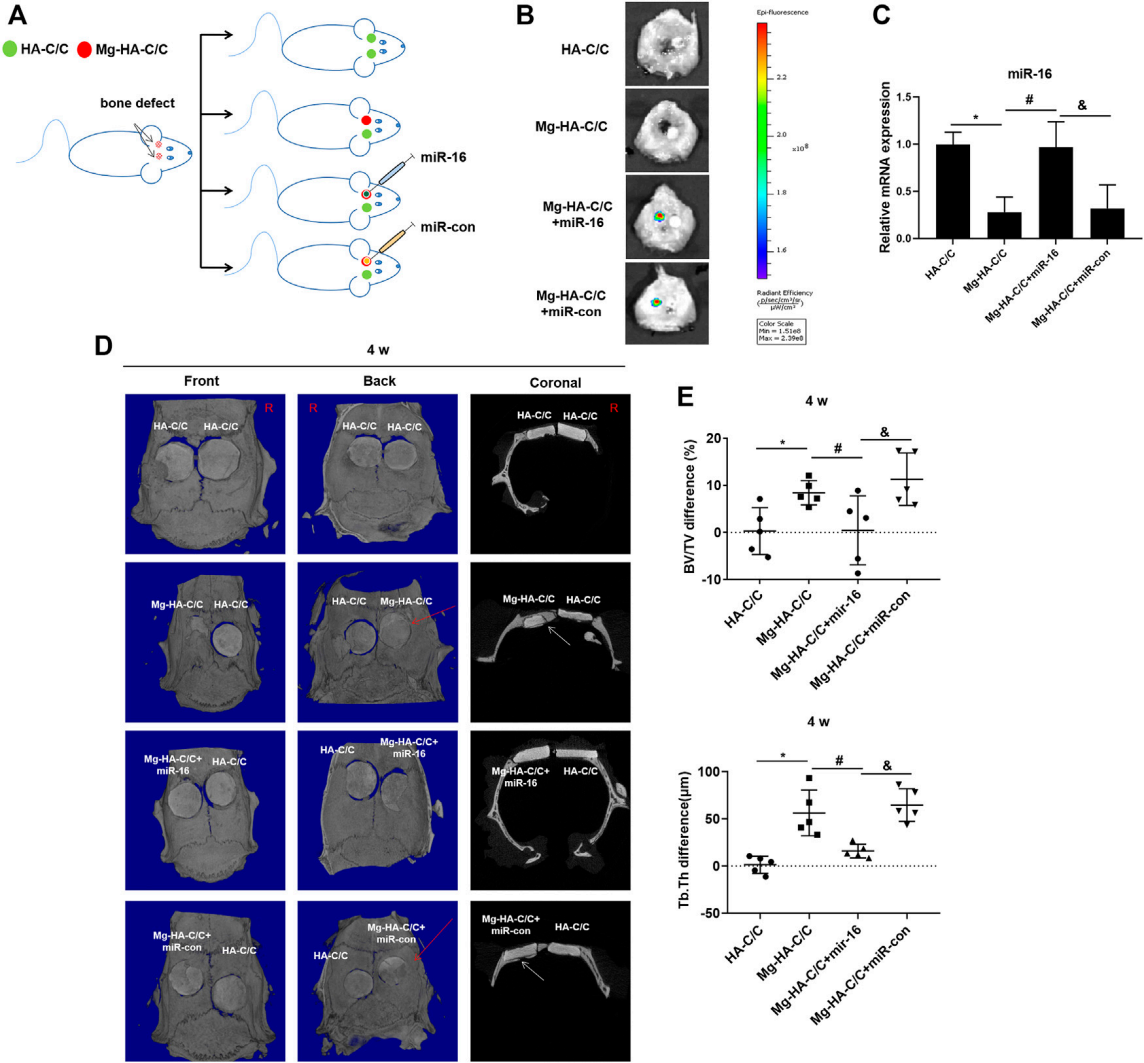
Overexpression of miR-16 blocks the enhancement of bone formation and reconstruction induced by Mg-HA-C/C composites at 4 weeks after implantation. The HA-C/C, Mg-HA-C/C, Mg-HA-C/C + miR-16, or Mg-HA-C/C + miR-con composites were implanted on the drilled skulls, and the rats were maintained for 4 weeks. **(A)** Schematic graph illustrating the animal experiment. **(B)** The transfection efficiency was assessed by a visible light imaging system. **(C)** The levels of miR-16 in the skull were assessed by qRT-PCR (mean ± SD, *n* = 6). **(D)** The 3D-reconstructed micro-CT images and coronal images acquired. The red arrow on 3D reconstruction and the white arrows on coronal images indicate new bone tissue. **(E)** Values for BV/TV and Tb.Th were calculated (mean ± SD, *n* = 5), ^*, #, &^ *p* < .05.

**FIGURE 7:**
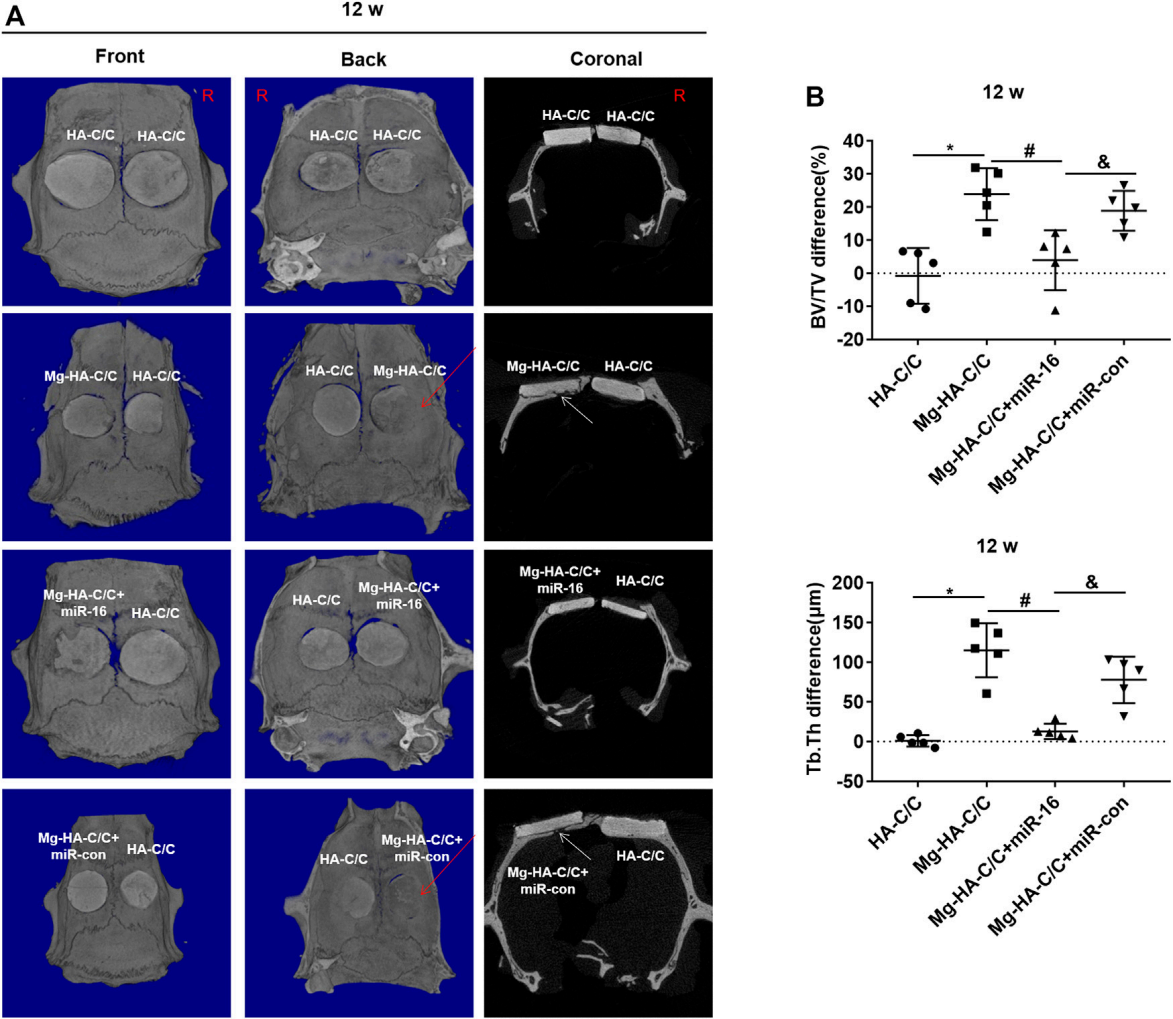
Overexpression of miR-16 blocks the enhancement of bone formation and reconstruction induced by Mg-HA-C/C composites at 12 weeks after implantation. The HA-C/C, Mg-HA-C/C, Mg-HA-C/C + miR-16, or Mg-HA-C/C + miR-con composites were implanted on the drilled skulls, and the rats were maintained for 12 weeks. **(A)** 3D-Reconstructed micro-CT images and coronal images acquired at 12 weeks after implantation. The red arrows on 3D reconstructions and the white arrows on coronal images indicate new bone tissue. **(B)** Values for BV/TV and Tb.Th were calculated by CTAn analysis software (mean ± SD, *n* = 5). ^*, #, &^ *p* < .05.

To determine if miR-16 is involved in the promotion of bone healing by the Mg-HA-C/C composite, a lentiviral vector was injected *in situ* into the bone defects implanted with the Mg-HA-C/C composite (Figure 6A). Fluorescence imaging of rat skulls showed expression of GFP at the injection site, which indicated that the lentivirus transfection was effective (Figure 6B). At 4 weeks after the operation, Mg-HA-C/C composites promoted bone formation more than those with pure HA coatings. However, after *in situ* injection of an mir-16 mimic lentivirus vector, the promoting effect induced by Mg-HA-C/C composites was blocked (Figure 6C). Quantitative analysis demonstrated that the percentages of newly formed bone volume (BV/TV) and trabecular thickness (Tb.Th) for the Mg-HA-C/C group were higher than those in the group treated with the pure HA-C/C composite, an effect that was blocked by the miR-16 mimic (Figures 6D,E). At 12 weeks after the implantation, the results were consistent with the results acquired at 4 weeks, but the effect was stronger (Figure 7).

**FIGURE 8:**
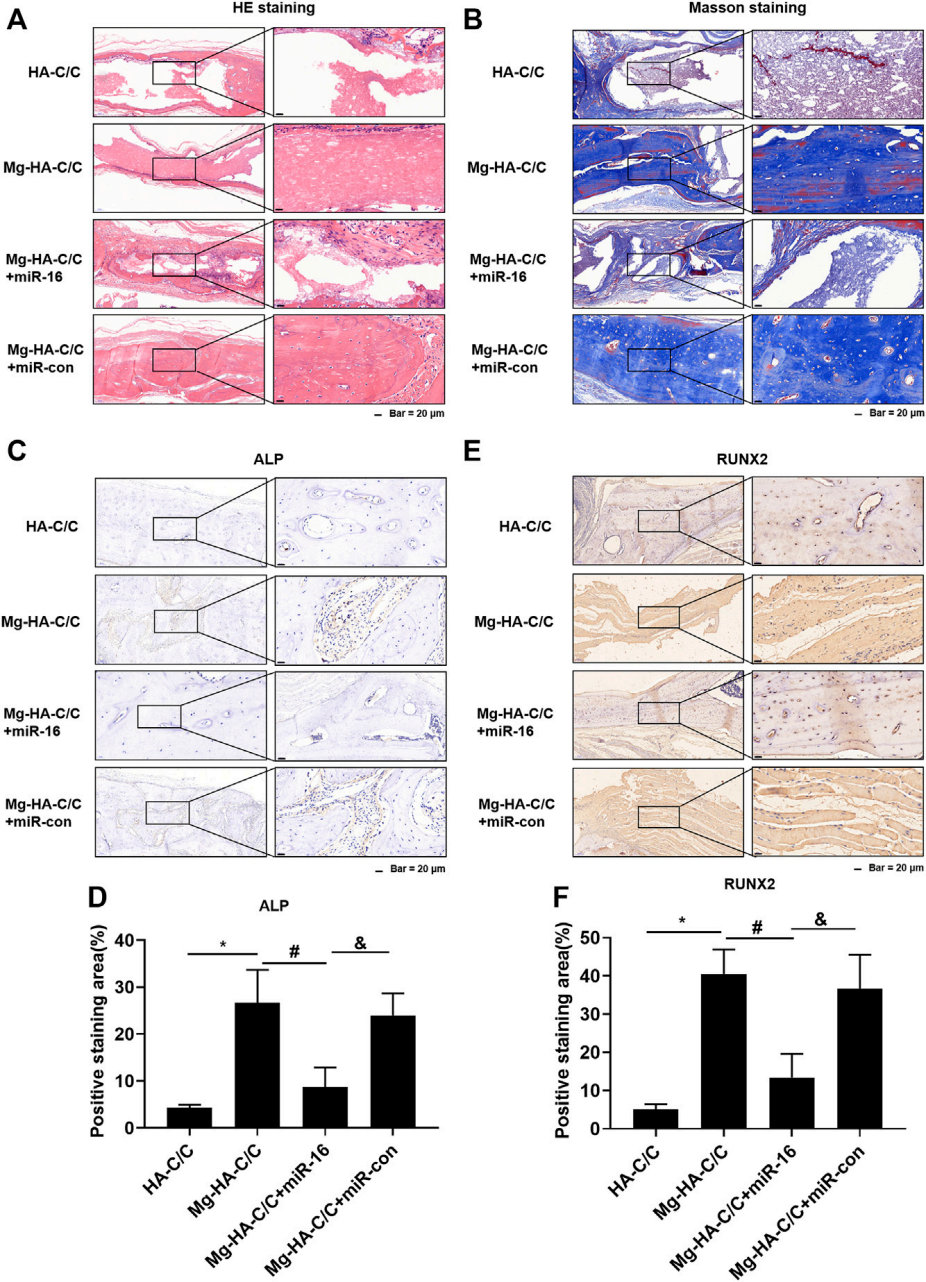
Overexpression of miR-16 blocks the promotion of bone formation and the increases of ALP and RUNX2 levels induced by Mg-HA-C/C composites. The HA-C/C, Mg-HA-C/C, Mg-HA-C/C + miR-16, or Mg-HA-C/C + miR-con composites were implanted on the drilled skulls, and the rats were maintained for 12 weeks. HE staining **(A)** and Masson staining **(B)** of the skull were performed. **(C)** IHC staining of ALP was performed, and **(D)** the positive staining areas were quantified. **(E)** IHC staining of RUNX2 was accomplished, and **(F)** the positive staining areas were quantified (mean ± SD, *n* = 5). ^*, #, &^ *p* < .05.

Qualitative histological results are shown in Figure 8. At week 12 after implantation, HE and Masson staining of skulls treated with Mg-HA-C/C composites were stronger than that in skulls treated with HA-C/C composites; however, this effect was blocked by overexpression of miR-16 (Figures 8A,B). Immunostainings for RUNX2 and ALP in the group treated with Mg-HA-C/C composites were higher, an effect that was inhibited by the miR-16 mimic (Figures 8C–F). These results suggest that Mg ions form Mg-HA-C/C composites promote repair and reconstruction of bone defects through via inhibiting miR-16.

### 3.5 Biosafety Evaluation of *in vivo* Experiments

Since the biological material is to be implanted in humans, we must ensure its safety. To evaluate the biological safety of Mg-HA-C/C composites, we collected the whole blood and serum of rats and assessed the hematological (Supplementary Figure S1) and blood biochemical indexes (Supplementary Figure S2). For each group, there was no statistical difference in hematological and blood biochemical indexes (RBC, WBC, Hgb, Hct, PLT, ALT, AST, ALP, urea, and creatinine). Therefore, for these assessments, the Mg-HA-C/C composites have no measureable side effects.

## 4 Discussion

Bone defects are common in active young people, but they are difficult to heal due to their poor regeneration capacity and to complex and inherent hierarchical structures (Castro, O'Brien and Zhang, 2015). A variety of bone graft substitute materials have been developed, with the objective that these materials would have better biocompatibility and a stronger promoting effect on bone formation (Jiang et al., 2020; Mukherjee et al., 2020; Stahl and Yang, 2020). In our research, we synthesized Mg-HA coatings on C/C composites. The results show that Mg-HA-C/C composites release Mg ions, and Mg-HA coatings have better biological and mechanical properties than pure HA coatings and exert stronger promotion of bone regeneration.

Mg ions, which support and sustain health and life, are essential for the human body (de Baaij, et al., 2015). These ions participate in numerous biological mechanisms; as examples, they are involved in the regulation of ion channels, DNA stabilization, enzyme activation, and stimulation of cell growth and proliferation (Nabiyouni et al., 2018). Mg-containing materials have a promoting effect on bone formation (Bedair et al., 2020; Ma et al., 2020; Wang et al., 2020; Cheon et al., 2021). Mg-HA, with its lower microstructural anisotropy and less deformation twinning, improves the grain structure and reduces the crystallographic texture of composites, although their compressive stress is lower (Xin-Ye et al., 2014).

In our present study, after adding Mg to HA, the surface morphology of the material was changed. Surface topography affects the pattern of gene expression of bone-related proteins (OSP, OSN, bone sialoprotein, type I collagen, and ALP) (Thalji and Cooper, 2013). ALP is an enzyme that acts as a mineralization promoter (Sutthavas et al., 2021). RUNX2 is a marker in the first stage of bone formation, in which mesenchymal precursors commit to osteoblast differentiation lineage (Yuan et al., 2020). Sp7, which functions downstream of RUNX2 in osteogenesis, is necessary for the differentiation and function of mature osteoblasts (Nakashima et al., 2002). OPN and OCN are indicators of middle- to late-stage bone formation in which there is an increase of metabolic activity; bone cells deposit and mineralize the matrix (Jensen et al., 2010). As shown by our results, smaller particles are conductive to cell differentiation and bone regeneration. BMSCs were seeded on the surface of HA coatings with various Mg contents. BMSCs on the surface of Mg-HA coatings were denser, and their capacity for osteogenic differentiation was enhanced. The expressions of osteogenic markers (ALP, RUNX2, Sp7, OCN, and OPN) were increased with more extensive mineralization.

The addition of Mg to bone graft materials promotes healing of bone tissue. For example, a Mg/Ti hybrid system was used for fracture fixation (Tian et al., 2018), and Mg-strontium scaffolds were used for bone regeneration of critical-size segmental defects (Wang W. et al., 2019). In our animal experiments, compared with pure HA, Mg-HA promoted bone regeneration better. The results of micro-CT showed that bone tissue regeneration was more obvious after Mg-HA-C/C coatings were implanted. In this experiment, the regeneration of bone tissue inside the skull was more obvious than that of the outside. HE and Masson staining indicated that the Mg-HA coating on C/C composites promoted bone regeneration and healing. Immunohistochemical analysis also showed that Mg-HA-C/C composites increased the levels of RUNX2 and ALP, which are makers of bone formation.

In our previous study, we confirmed that Mg ions promote osteogenic differentiation of BMSCs by down-regulation of miR-16 (Qi, et al., 2020). Therefore, we designed animal experiments to determine if the Mg-HA-C/C composites also promoted bone formation and healing by regulating the mechanism of mR-16. A lentiviral vector of an miR-16 mimic was synthesized and injected it into the transplantation site of the Mg-HA-C/C composites. The miR-16 mimic blocked the increased values of BV/TV and Tb.Th and the higher levels of ALP and RUNX2 induced by Mg-HA-C/C composites, suggesting that miR-16 is involved in the promotion of bone formation and healing induced by Mg-HA-C/C composites.

The biosafety of the graft material is a relevant issue (Rohaizad et al., 2020). After being transplanted into the body, the material degrades, enters the circulatory system, and is metabolized in various tissues, a process that may produce toxic effects (Xia et al., 2020). In the present study, we collected blood and serum from rats to evaluate the toxicological characteristics of Mg-HA. There was no apparent abnormality in blood or in liver and kidney function (RBC, WBC, Hgb, Hct, Plt, ALT, AST, ALP, urea, and creatinine), indicating that, in animals, Mg-HA-C/C composites have little or no toxic effect. Thus, the present study verifies the effect of the Mg-HA-C/C composite on bone regeneration and reveals its possible molecular mechanism in animal models. In this preliminary investigation, the biological safety of Mg-HA-C/C composite was evaluated, and no obvious toxic effect was found.

## Conclusion

**FIGURE 9:**
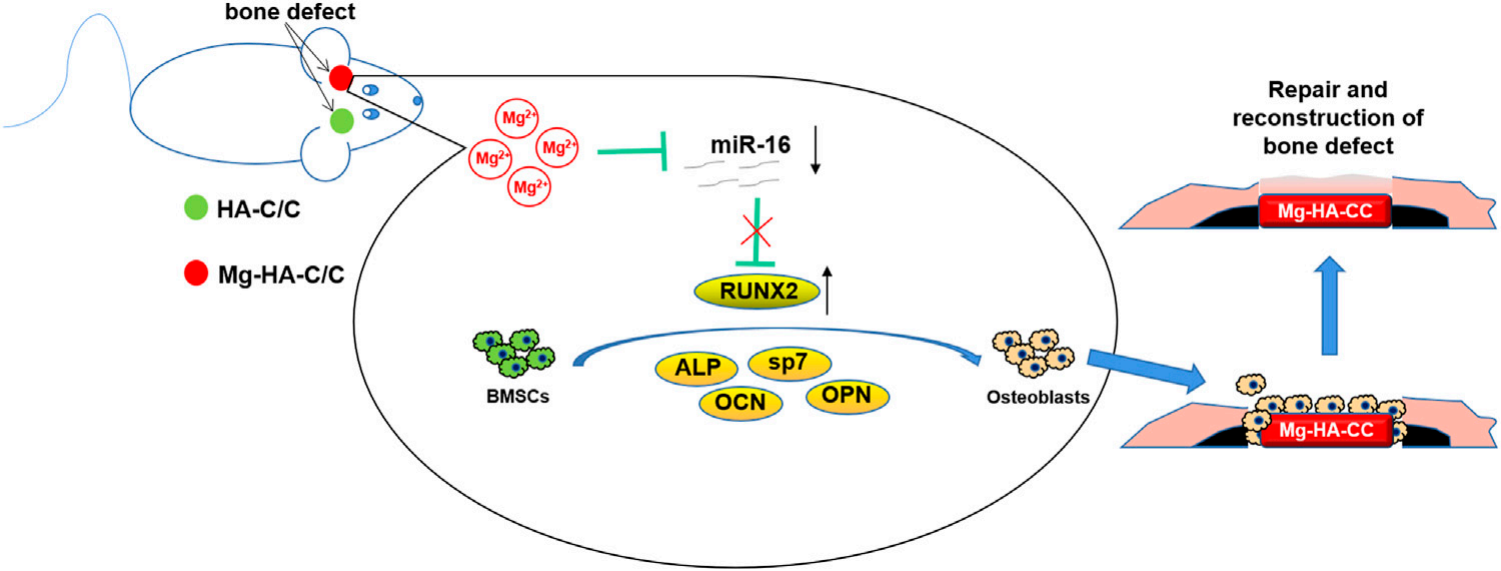
Schematic illustration of Mg-HA-C/C composites promoting bone healing and the mechanism in rats. The Mg-HA-C/C composites release Mg ions, which decrease levels of miR-16 and increase the expression of the osteogenesis-related gene, *RUNX2*, thus promoting the osteogenic differentiation of BMSCs and enhancing the regeneration of bone tissue.

In conclusion, we fabricate Mg-HA coating on C/C composites, which enhance osteogenesis differentiation of BMSCs and promote repair and regeneration of cartilage and subchondral bone in osteochondral defects via inhibiting miR-16. Although these composites show a promising prospect for bone repair and formation, further research should lead to improvements (Figure 9).

## Data Availability

The data used to support the findings of this study are available from the corresponding author upon request.

## Abbreviations

ALT, alanine aminotransferase; AST, aspartate aminotransferase; ALP, alkaline phosphatase; BMSCs, bone marrow mesenchymal stem cells; BMP-2, bone morphogenetic protein-2; BV/TV, bone volume to total volume; C/C Carbon/carbon; CREA, creatinine; HA, hydroxyapatite; Hgb, hemoglobin; Hct, hematocrit; Mg, magnesium; miRNAs, MicroRNAs; OCN, osteocalcin; OPN, osteopontin; PLT, platelets; RBC, red blood cells; Runx 2, runt-related transcription factor 2; Sp7, Osterix; Tb.Th, trabecular thickness; VEGF, vascular endothelial growth factor; WBC, white blood cells.
