# Supplementary material for: Mg-HA-C/C Composites Promote Osteogenic Differentiation and Repair Bone Defects Through Inhibiting miR-16
Source: Front Bioeng Biotechnol. 2022 Feb 4;10:838842. doi: 10.3389/fbioe.2022.838842 (PMC8854763; doi:10.3389/fbioe.2022.838842)
Supplement: Supplementary file 1 [file DataSheet1.zip › Supplementary files/Supplementary materials.docx]

**Supplementary materials**

**Supplementary methods**

***Systemic toxicological profile***

Blood samples were collected into EDTA-containing tubes for hematological and genotoxicity studies or collected into serum separator gel tubes for biochemical studies. Hematological parameters, including red blood cells (RBC), white blood cells (WBC), hemoglobin (Hgb), hematocrit (Hct), and platelets (PLT) were determined by an automated hematology analyzer (Coulter T540 hematology system; Fullerton, CA, USA). The blood samples for biochemical analyses were centrifuged at 3000 rpm for 10 min; the serum was collected and analyzed by an automated analyzer Cobas® 6000 (Roche Diagnostics, Mannheim, Germany) for determination of alanine aminotransferase (ALT), aspartate aminotransferase (AST), alkaline phosphatase (ALP), urea, and creatinine (CREA).

**Supplementary Table**

**Supplementary Table S1.** The concentrations of Mg ions in the DMEM medium (mg/L, mean ± SD, n=3)

| Mg content of Mg-HA-C/C | Time (d) | | | |
| --- | --- | --- | --- | --- |
|  | 0 | 1 | 3 | 6 |
| 0 % | 20.78±1.56 | 20.72±1.18 | 20.78±2.07 | 20.45±1.43 |
| 10 % | 21.48±1.06 | 58.11±1.61^*^ | 62.67±2.37^*^ | 66.20±3.93^*^ |
| 20 % | 21.52±0.70 | 96.16±1.99^*, #^ | 114.38±2.41^*, #^ | 116.22±4.84^*, #^ |

**p* < 0.05 different from Mg-HA-C/C with 0 % Mg; #*p* < 0.05 different from Mg-HA-C/C with 10 % Mg.

**Supplementary figures and figure legends**


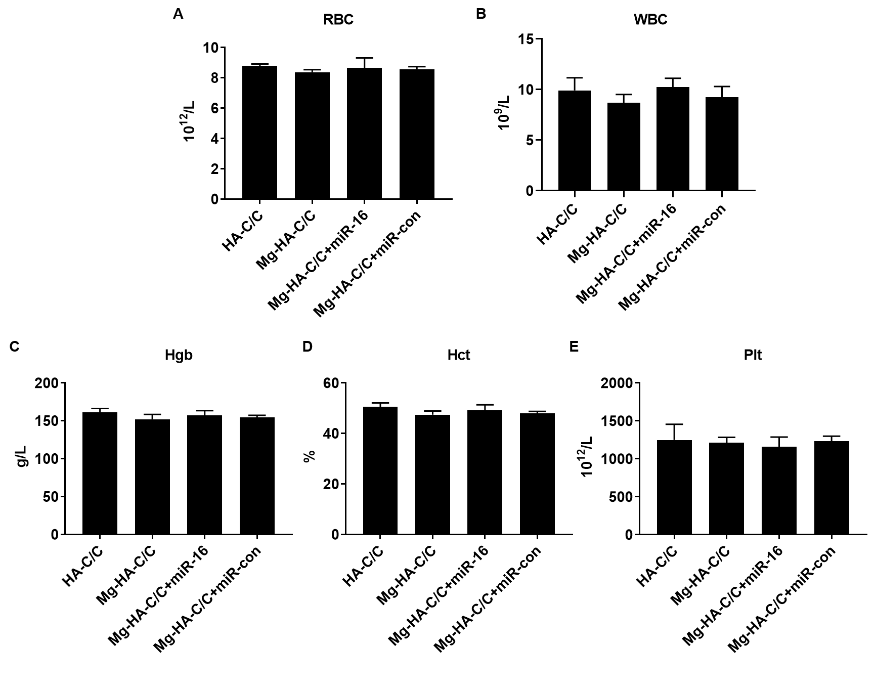


***Supplementary Figure S1. Biological effects of various materials as detected by hematological tests.***

The whole blood of rats was collected for biological safety assessment by hematological tests. (A-E) Hematological indexes for red blood cells (RBC), white blood cells (WBC), hemoglobin (Hgb), hematocrit (Hct) and platelets (PLT) were determined (mean ± SD, n=5).


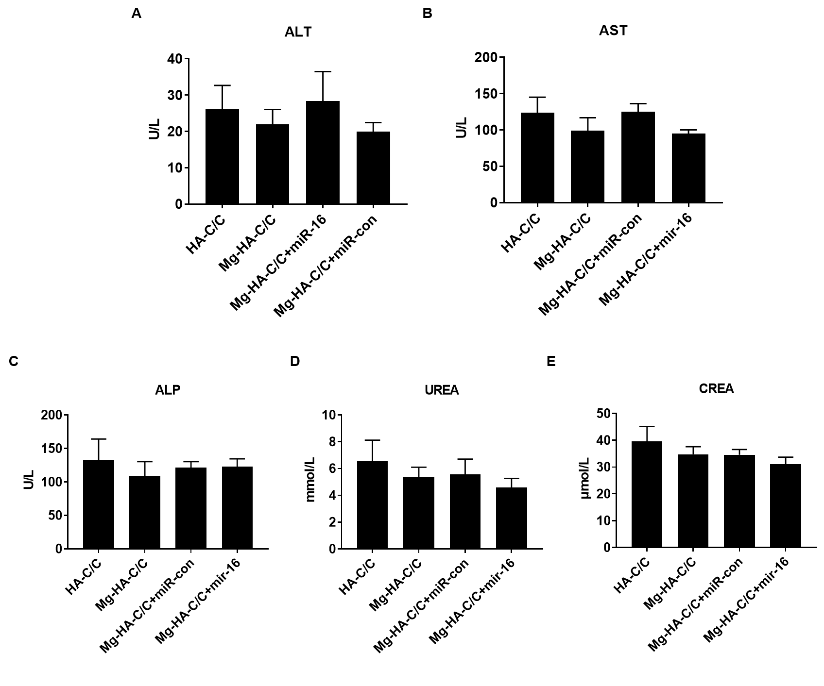


***Supplementary Figure S2. Biological effects of various materials as measured by blood biochemistry.***

The sera of rats were collected to determine the biochemical indexes. (A-E) alanine aminotransferase (ALT), aspartate aminotransferase (AST), alkaline phosphatase (ALP), urea, and creatinine (CREA) (mean ± SD, n=5).

***Supplementary Video 1: Video of bone formation as seen from various angles.***
