# Supplementary figures and images for: Mg-HA-C/C Composites Promote Osteogenic Differentiation and Repair Bone Defects Through Inhibiting miR-16
Source: Front Bioeng Biotechnol. 2022 Feb 4;10:838842. doi: 10.3389/fbioe.2022.838842 (PMC8854763; doi:10.3389/fbioe.2022.838842)

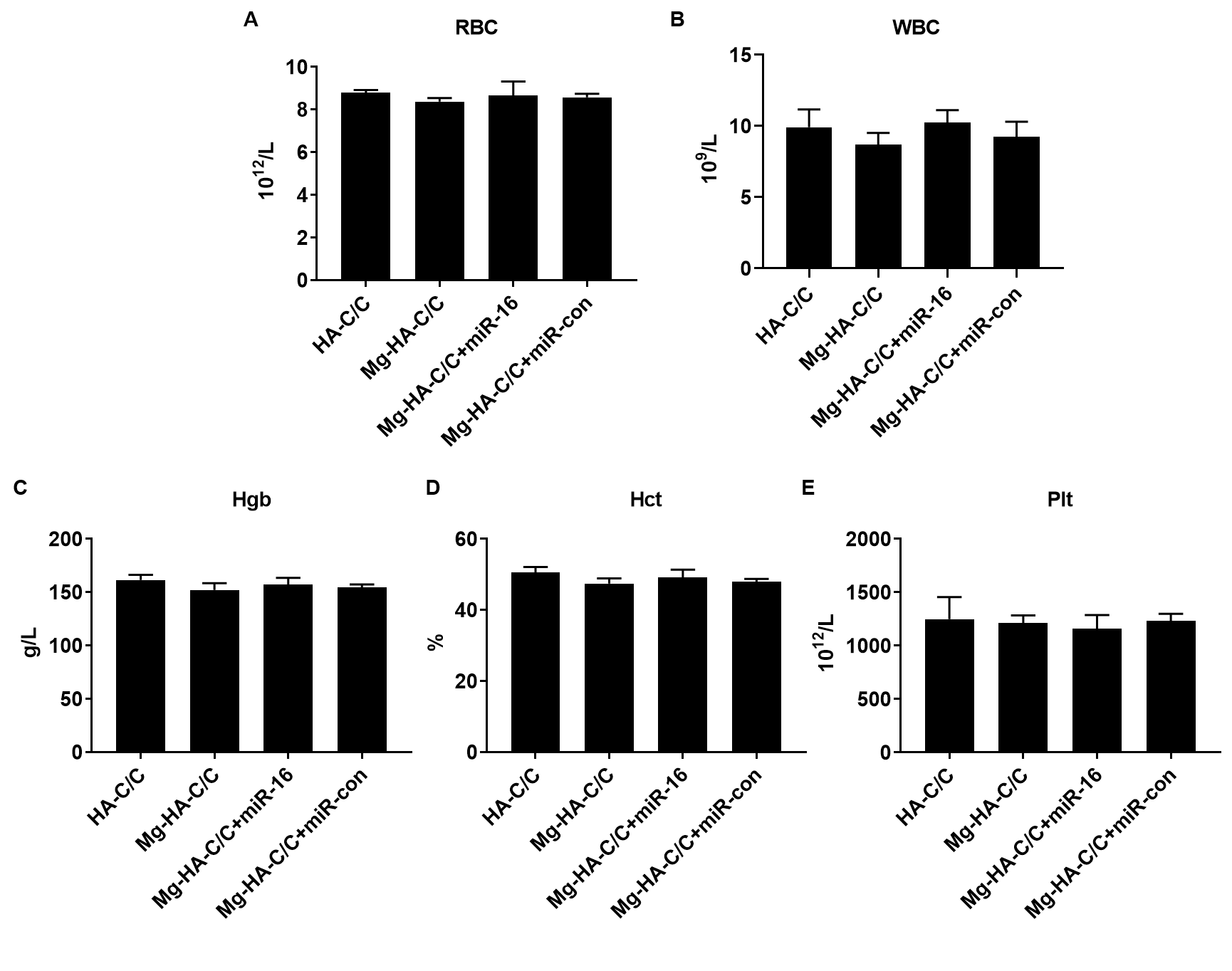

Supplement: Supplementary file 1 [file DataSheet1.zip › Supplementary files/Supplementary Figure S2.tif]

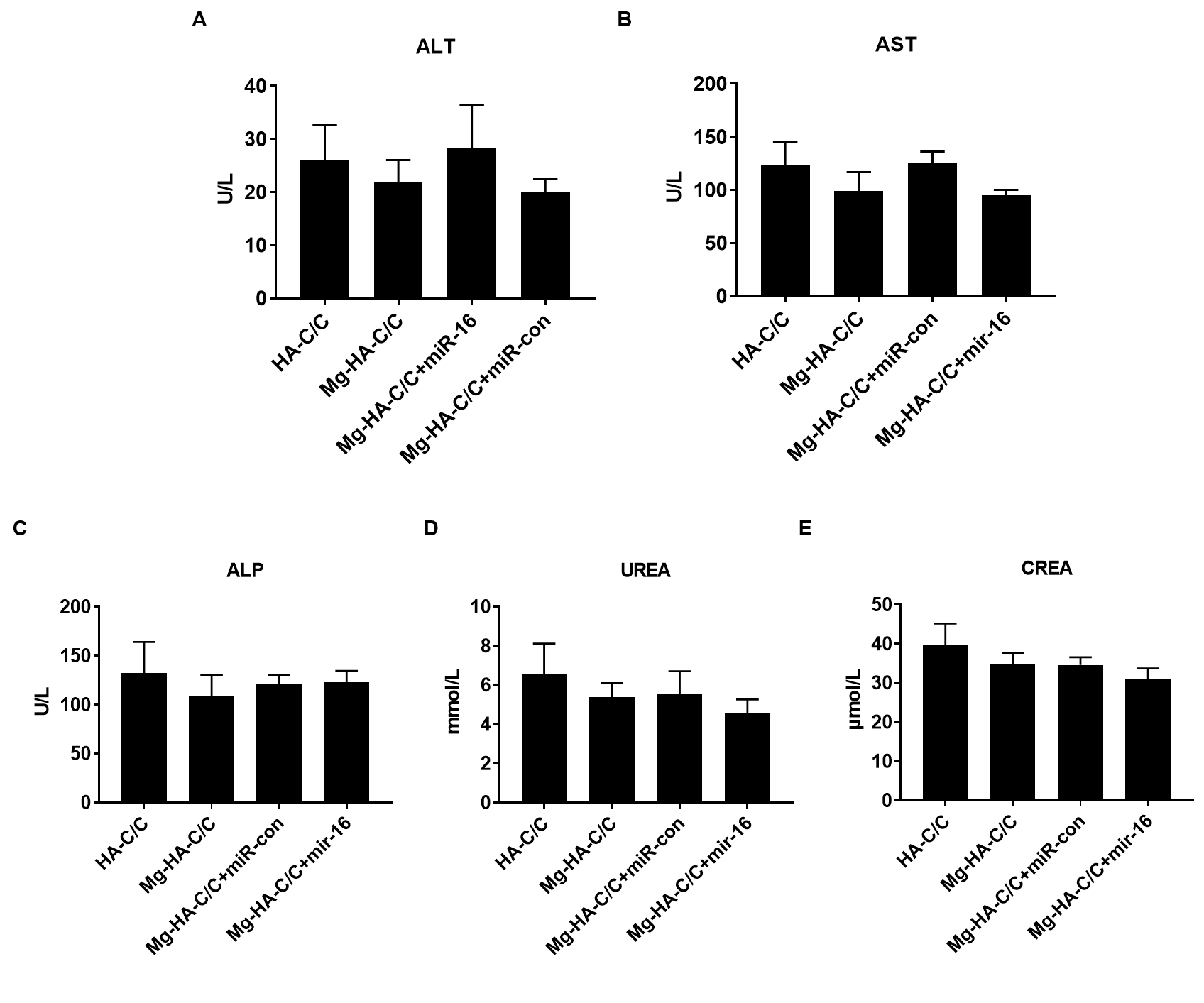

Supplement: Supplementary file 1 [file DataSheet1.zip › Supplementary files/Supplementary Figure S3.tif]
